# Supplementary material for: Controlled Delivery of an Anti-Inflammatory Toxin to Macrophages by Mutagenesis and Nanoparticle Modification
Source: Nanomaterials (Basel). 2022 Jun 23;12(13):2161. doi: 10.3390/nano12132161 (PMC9268525; doi:10.3390/nano12132161)
Supplement: Supplementary file 1 [file nanomaterials-12-02161-s001.zip › nanomaterials-1755911-supplementary.pdf]

Supplementary Material

Controlled Delivery of an Anti-Inflammatory Toxin to Macrophages by Mutagenesis and Nanoparticle Modification

Ayaka Harada [1,†], Hiroyasu Tsutsuki [2,†], Tianli Zhang [2], Kinnosuke Yahiro [3], Tomohiro Sawa [2] and Takuro Niidome [1,*]

1 Faculty of Advanced Science and Technology, Kumamoto University, 2-39-1 Kurokami, Chuo-ku, Kumamoto 860-8555, Japan; 201d8604@st.kumamoto-u.ac.jp
2 Department of Microbiology, Graduate School of Medical Sciences, Kumamoto University, 1-1-1 Honjo, Chuo-ku, Kumamoto 860-8556, Japan; tsutsuki@kumamoto-u.ac.jp (H.T.); zhangtianli220@hotmail.com (T.Z.); sawat@kumamoto-u.ac.jp (T.S.)
3 Department of Microbiology and Infection Control Sciences, Kyoto Pharmaceutical University, 5 Misasagi-Nakauchi-cho, Yamashina-ku, Kyoto 607-8414, Japan; kin816tas@mb.kyoto-phu.ac.jp
* Correspondence: niidome@kumamoto-u.ac.jp
† These authors contributed equally to this work.

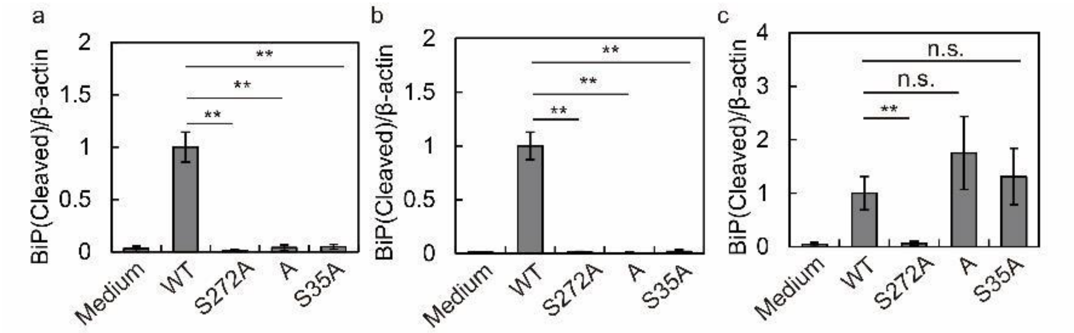

Figure S1. Densitometry of BiP cleavage in (**a**) J774.1 cells, (**b**) HeLa cells, and (**c**) a HeLa cell lysate. The cells and cell lysate were treated with 5 μg/mL toxin for 1 h. After treatment, the cells and cell lysate were incubated in SDS sample buffer and then subjected to Western blotting and densitometry. Data are expressed as means ± S.D. (n = 3). Statistical analyses were performed using Student's t-test. n.s., not significant. ***p < 0.01.*

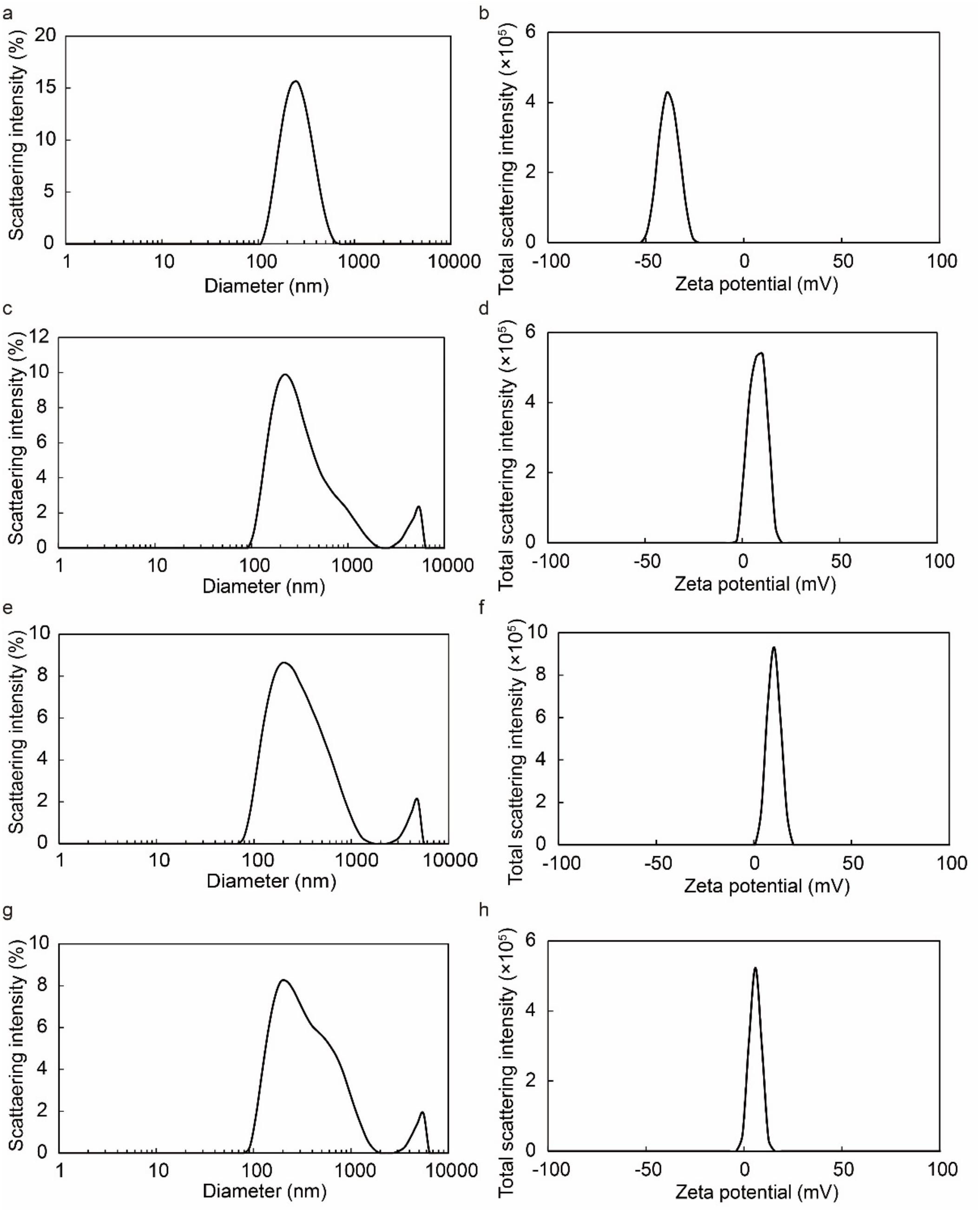

Figure S2. Characterization of PLGA NPs. Dynamic light scattering of (**a**) PLGA NPs, (**c**) WT-PLGA NPs, (**e**) A-PLGA NPs and (**g**) S35A-PLGA NPs in water. Zeta potentials of (**b**) PLGA NPs, (**d**) WT-PLGA NPs, (**f**) A-PLGA NPs and (**h**) S35A-PLGA NPs were measured at room temperature.

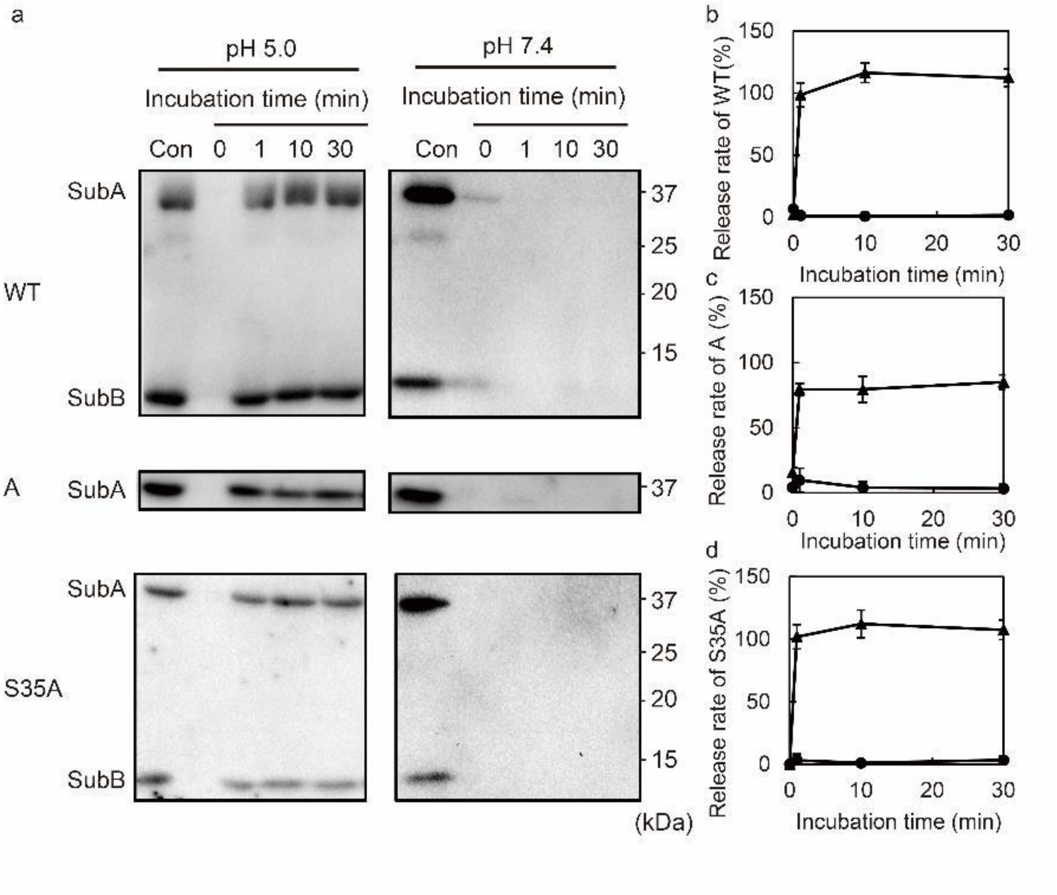

Figure S3. pH-responsive dissociation of His-tagged WT, A, and S35A from PLGANPs. (**a**) Toxin-modified PLGA NPs were treated for 0–30 min with buffers at the indicated pH value. After centrifugation, the supernatant was analyzed by Western blotting and densitometry. Quantification of released (**b**) WT, (**c**) A, and (**d**) S35A by densitometry (●: pH 5.0; ▲: pH 7.4). Data are expressed as means ± S.D. (n = 3).

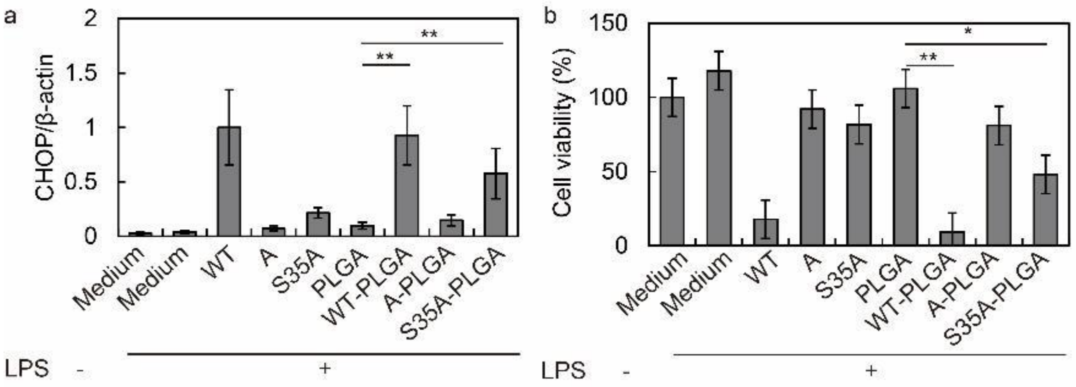

Figure S4. Evaluation of ER stress and cytotoxicity in J774.1 cells. The cells were treated with or without LPS (100 ng/mL) in the presence 5 μg/mL toxin and 20 μg/mL PLGA NPs for 8 and 24 h. After 8 h, the cells were lysed in SDS sample buffer and then subjected to Western blotting and densitometry. Additionally, the cells were subjected to MTT assays at 24 h of treatment. (**a**) Densitometry of CHOP expression at 8 h. (**b**) Evaluation of cell viability by MTT assays. Data are expressed as means ± S.D. (n = 3). Statistical analyses were performed using Student's t-test. $^{*}p < 0.05$, $^{**}p < 0.01$.

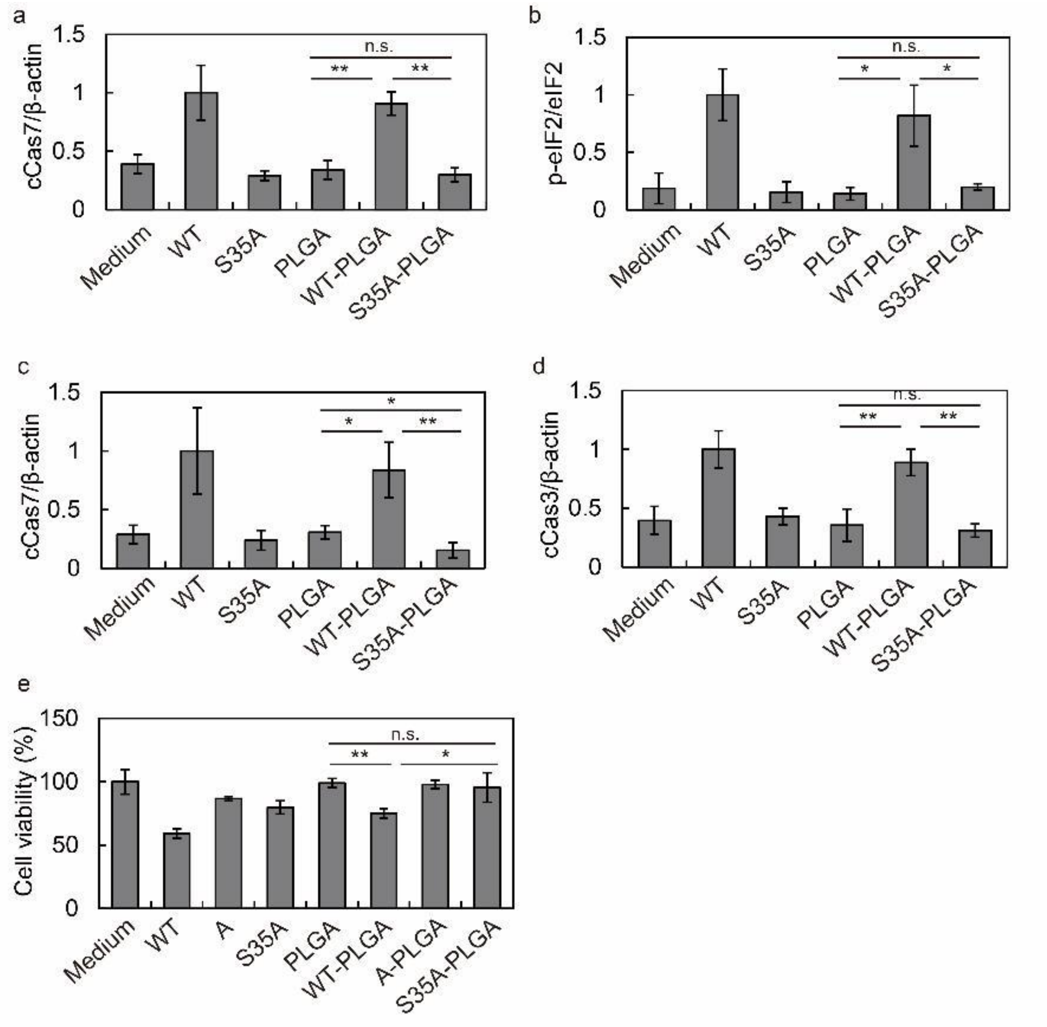

Figure S5. Evaluation of ER stress and apoptosis induction in HeLa cells. The cells were treated with or without 5 μg/mL toxin and 20 μg/mL PLGA NPs for 3, 8, and 24 h. After 3 and 8 h, the cells were lysed in SDS sample buffer and subjected to Western blotting and densitometry. Additionally, cell viability was evaluated by MTT assays at 24 h of treatment. (**a**) Densitometry of caspase 7 activation at 3 h. (**b**) Densitometry of eIF2 phosphorylation at 3 h. (**c**) Densitometry of caspase 7 activation at 8 h. (**d**) Densitometry of caspase 3 activation at 8 h. (**e**) Evaluation of cell viability by MTT assays. Data are expressed as means ± S.D. (n = 3). Statistical analyses were performed using Student's t-test. n.s., not significant. $*p < 0.05$, $**p < 0.01$.
